# Supplementary figures and images for: Sociodemographic and behavioral factors associated with diet quality among low-income community health center patients with hypertension
Source: PLoS One. 2025 Jan 13;20(1):e0299781. doi: 10.1371/journal.pone.0299781 (PMC11730379; doi:10.1371/journal.pone.0299781)

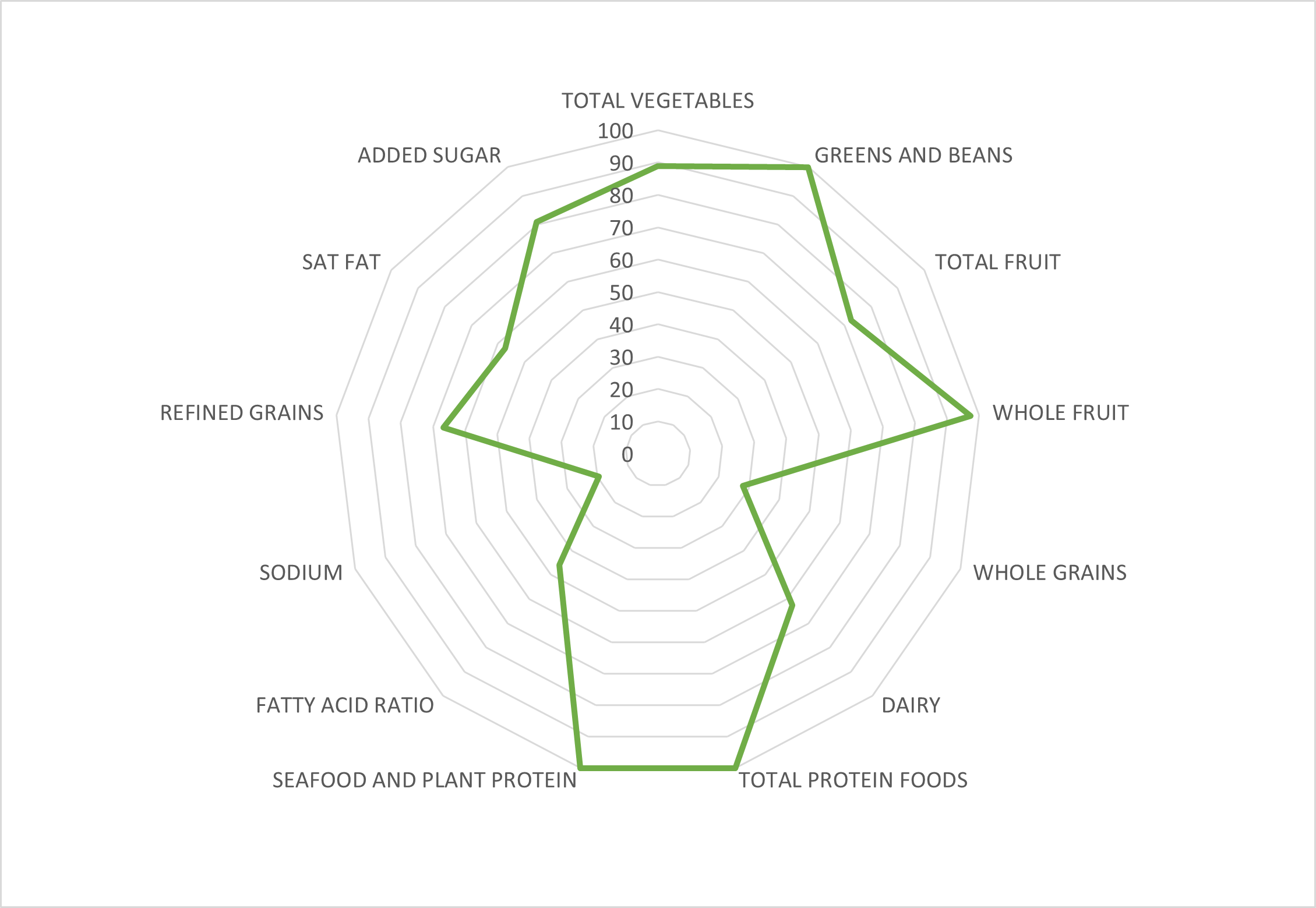

Supplement: S1 Fig — Note: Scores touching the outer ring represent the maximum score for a subcomponent (100% of the maximum score). A perfect diet quality score of 100 would be represented by touching the outer ring for all subcomponents. (TIF) [file pone.0299781.s001.tif]

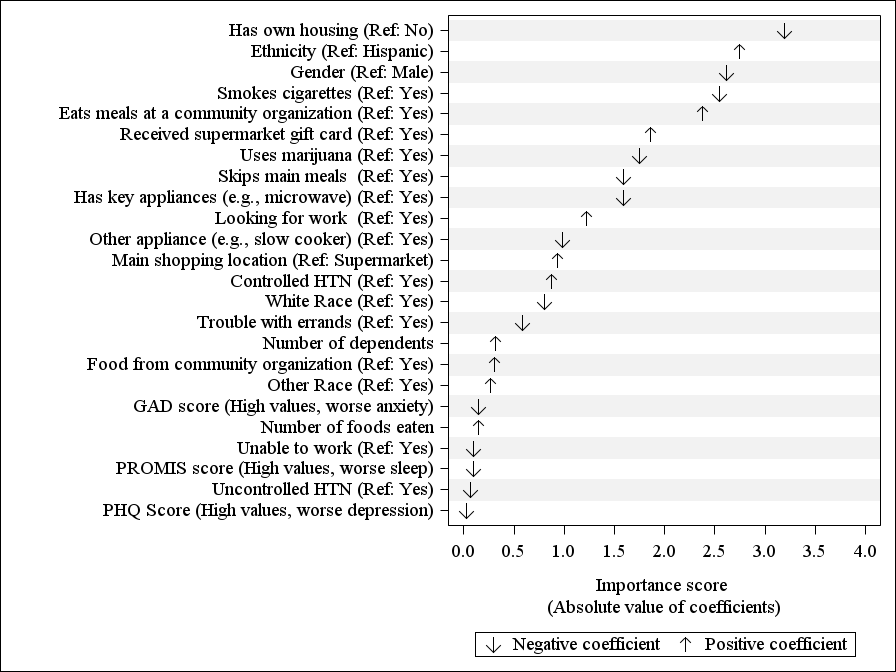

Supplement: S2 Fig — Note: GAD, General Anxiety Disorders Questionnaire; PHQ, Patient Health Questionnaire; PROMIS, Patient-Reported Outcomes Measurement Information System Sleep Disturbance Questionnaire. Factors are ranked from top to bottom in order of importance based on the absolute value of the model coefficients. A positive coefficient means that the reference value is associated with better diet quality. A negative coefficient means that the reference value is associated with worse diet quality. (TIF) [file pone.0299781.s002.tif]

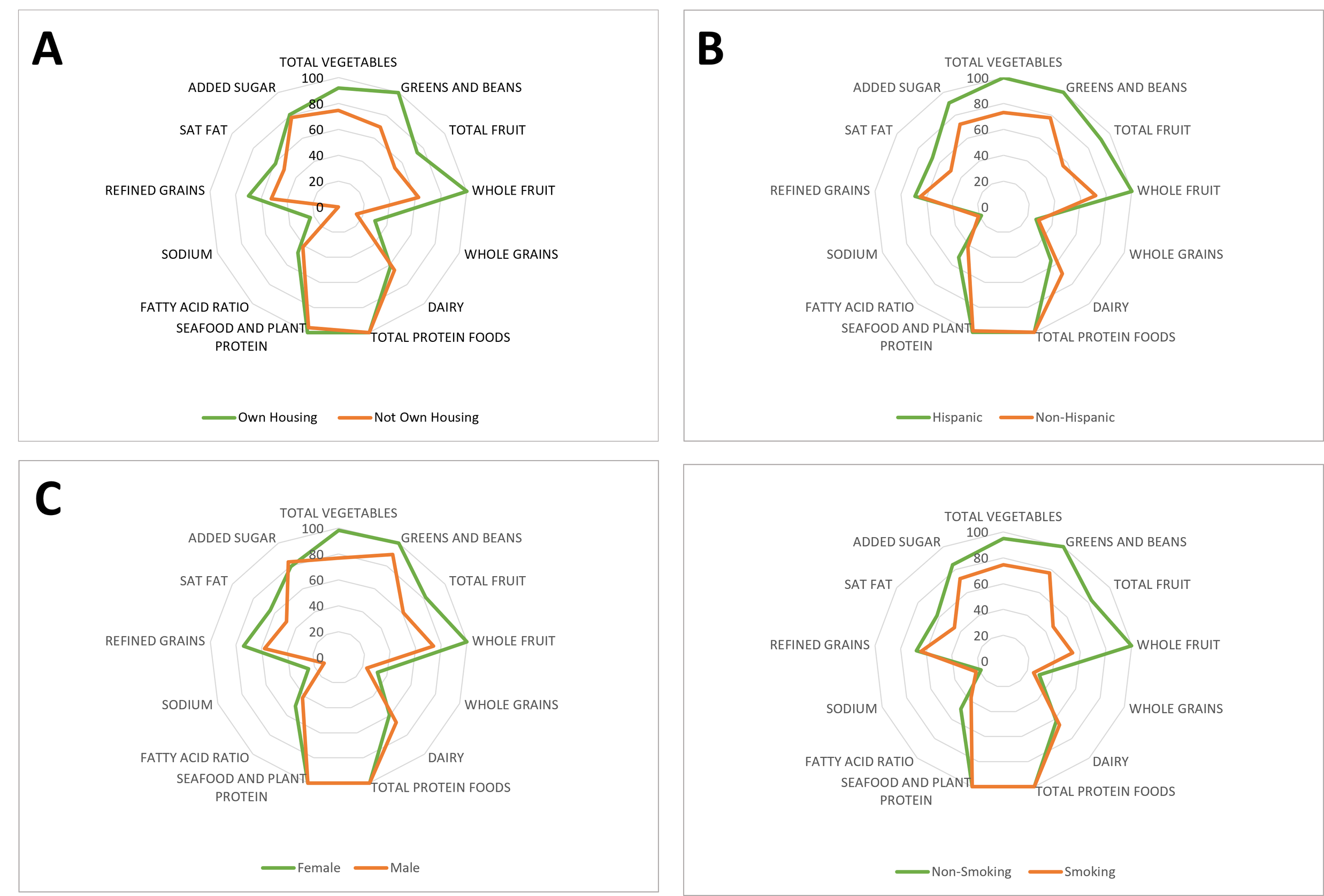

Supplement: S3 Fig — Note: A = HEI-2020 Total Scores by Housing Situation; B = HEI-2020 Total Scores by Ethnicity; C = HEI-2020 Total Scores by Gender; D = HEI-2020 Total Scores by Smoking; Total Scores by ethnicity. Scores touching the outer ring represent the maximum score for a subcomponent (100% of the maximum score). A perfect diet quality score of 100 would be represented by touching the outer ring for all subcomponents. (TIF) [file pone.0299781.s003.tif]
