# Supplementary material for: Sociodemographic and behavioral factors associated with diet quality among low-income community health center patients with hypertension
Source: PLoS One. 2025 Jan 13;20(1):e0299781. doi: 10.1371/journal.pone.0299781 (PMC11730379; doi:10.1371/journal.pone.0299781)
Supplement: S1 Table — Note: There were 0 participants with missing values for all other variables included in modeling. (DOCX) [file pone.0299781.s004.docx]

**Supplemental Table 1: Number of Participants with Missing Data for Factors Including in the LASSO model**

| **Variable** | **Number Missing** |
| --- | --- |
| Race | 8 |
| Marital status | 6 |
| Income | 8 |
| Education | 1 |
| Employment status | 2 |
| Household size | 2 |
| Number of Dependents | 5 |
| Smoking Status | 3 |
| Drug abuse (i.e., illicit or prescription drugs) | 1 |
| Worry about housing | 5 |
| Moved in the last 12 months | 2 |
| Housing Instability | 5 |
| Cost-related medication underuse: Trouble filling prescriptions | 2 |
| US Department of Agriculture (USDA) Food Security score | 5 |
| Perceived Stress Scale (PSS) score | 7 |
| Weekly Stress Inventory (WSIFSQ) score | 2 |
| Patient Health Questionnaire (PHQ-8) score | 5 |
| Generalized Anxiety Disorders (GAD-7) score | 5 |
| Patient-Reported Outcomes Measurement Information System (PROMIS) Sleep Disturbance score | 2 |
| Medical Outcomes Study Social Support Survey (MOS) score | 9 |
| Everyday Discrimination Scale (EDS) score | 2 |
| Have a freezer | 12 |
| Have other countertop cooking appliances (e.g., toaster oven, slow cooker, or electric grill) | 13 |
| Have other key appliances (e.g., refrigerator, microwave oven, stove, oven) | 14 |
| International Physical Activity Questionnaire (IPAQ): Walking (min/wk) | 33 |
| International Physical Activity Questionnaire (IPAQ): Moderate Activity (min/wk) | 24 |
| International Physical Activity Questionnaire (IPAQ): Vigorous Activity (min/wk) | 18 |
| International Physical Activity Questionnaire (IPAQ): Sitting (min/wk) | 54 |
| Received food assistance (i.e., Family or friends) | 1 |
| Received food assistance (i.e., Meals eaten at a community organization) | 1 |
| Received food assistance (i.e., Meals delivered to your home) | 1 |
| Received food assistance (i.e., Food from a community organization) | 1 |
| Received food assistance (i.e., Supermarket gift card) | 1 |
| Received food assistance (i.e., SNAP) | 1 |
| Received other food assistance (e.g., food from a health clinic, WIC, Other assistance) | 1 |

Note: There were 0 participants with missing values for all other variables included in modeling.
