# Supplementary material for: Sociodemographic and behavioral factors associated with diet quality among low-income community health center patients with hypertension
Source: PLoS One. 2025 Jan 13;20(1):e0299781. doi: 10.1371/journal.pone.0299781 (PMC11730379; doi:10.1371/journal.pone.0299781)
Supplement: S1 File — (DOCX) [file pone.0299781.s005.docx]

**Supplemental Information on Food, Housing, and Transportation Insecurity Survey Questions**

Food and Housing. This section will ask you some questions about your food and your home.

10. What type of store is the store where you buy most of your food? (Choose the best answer)

O Supermarket

O Small grocery store

O Corner store or convenience store

O Supercenter (like WalMart or Costco)

O Other : ______________________________

11. Next, here are several statements that people make about their food situations. For these statements, please choose whether the statement was often true, sometimes true, or never true for you in the last 12 months.

I worried whether my food would run out before I got money to buy more.

O Often true

O Sometimes true

O Never true

12. The food that I bought just didn't last and I didn't have money to get more.

O Often true

O Sometimes true

O Never true

13. I couldn't afford to eat balanced meals.

O Often true

O Sometimes true

O Never true

14. In the last 12 months, did you ever cut the size of your meals or skip meals because there wasn't enough money for food?

O Yes

O No

14a. How often did this happen?

O Almost every month

O Some months but not every month

O In only 1 or 2 months

15. In the last 12 months, did you ever eat less than you felt you should because there wasn't enough money for food?

O Yes

O No

16. In the last 12 months, were you ever hungry, but didn't eat, because there wasn't enough money for food?

O Yes

O No

17. In the last 12 months, did you lose weight because there wasn't enough money for food?

O Yes

O No

18. In the last 12 months did you ever not eat for a whole day because there wasn't enough money for food?

O Yes

O No

18a. How often did this happen?

O Almost every month

O Some months but not every month

O In only 1 or 2 months

19. What is your housing situation today?

O I am sleeping in my own home/apartment (rented or owned)

O I do not have my own housing (such as, I am staying with others, in a shelter, homeless, etc)

O Prefer not to answer

20. Where are you currently sleeping?

O A friend or family member's home

O A shelter

O Transitional housing (hotel or other temporary housing)

O Other: __________________________

O Outside (on the street, in a park, in a car, etc)

21. Think about the place you live or stay. Do you have any of the following problems?

(Check all that apply)

⬜ Pests such as bugs, ants, or mice

⬜ Mold

⬜ Lead paint or pipes

⬜ Lack of heat

⬜ Oven or stove not working

⬜ Smoke detectors missing or not working

⬜ Water leaks

⬜ received an eviction or foreclosure notice

⬜ am behind on paying mortgage or rent

⬜ None of the above

22. Which of these appliances do you have in your home to cook or store food?

|  | Yes | No |
| --- | --- | --- |
| 22a. Refrigerator | O | O |
| 22b. Freezer (attached to refrigerator or stand-alone) | O | O |
| 22c. Microwave oven | O | O |
| 22d. Stove | O | O |
| 22e. Oven | O | O |
| 22f. Other countertop cooking appliance (toaster oven, slow cooker, or electric grill) | O | O |

23. Are you worried that in the next 2 months you may not have your own housing to live in?

O Yes

O No

O Prefer not to answer

24. In the past 12 months, has the electric, gas, oil, or water company threatened to shut off services in your home?

O Yes

O No

O Already shut off

O Prefer not to answer

25. How many times have you moved in the past 12 months?

O Two or more times

O One time

O Zero (I did not move)

O Prefer not to answer

26. In the past 12 months, have you or your household received food or help buying food from any of the sources below?

(Check all that apply)

⬜Family or friends

⬜Meals eaten at a community organization, such as a community center, soup kitchen, shelter

⬜Meals delivered to your home (Community Servings, Meals on Wheels, etc)

⬜Food from a community organization, such as a food bank, food pantry, mobile truck, or church

⬜Food from your health clinic

⬜Supermarket gift card

⬜Food Stamps/EBT/Supplemental Nutrition Assistance Program (SNAP)

⬜Women, Infants, and Children (WIC) Program

⬜Other: _______________________________

⬜None

26a. In general, how difficult has it been in the past 12 months to get the help you needed to have enough food?

O Very difficult

O Somewhat difficult

O Somewhat easy

O Very easy

27. In the past 12 months, have you or your household received or done any of the following to help you with housing or having a place to live?

(Check all that apply)

⬜Lived or stayed with family or friends

⬜Stayed in a shelter or other temporary housing

⬜Financial support for rent or mortgage from family or friends

⬜Financial support for rent or mortgage from a community organization

⬜Housing voucher program (such as Section 8, or the Alternative Housing Voucher Program)

⬜Financial support for paying utility bills (such as gas, electricity, internet)

⬜Help with searching or applying for affordable housing

⬜Other: ______________________________

⬜None

27a. In general, how difficult has it been in the past 12 months to get the help you needed with housing?

O Very difficult

O Somewhat difficult

O Somewhat easy

O Very easy

28. In the past 12 months, have you used or received assistance from the following transportation-related services?

(Check all that apply.)

⬜MBTA's 'The RIDE'

⬜Reduced MBTA fares

⬜MART program

⬜Free transportation to or from appointments organized by my health clinic

⬜A free coupon, voucher, gift card, or Charlie Card to pay for a taxi or public transportation

⬜Another program for free or reduced-price transportation

⬜None

29. In the past 12 months, have any of the following people helped you with food, housing, or transportation needs?

(Check all that apply.)

⬜Doctor

⬜Nurse

⬜Social Worker

⬜Community Health Worker or Patient Navigator

⬜Case manager who helps with my chronic health conditions.

⬜Another person from the health center or hospital

⬜None
