# Supplementary material for: Sociodemographic and behavioral factors associated with diet quality among low-income community health center patients with hypertension
Source: PLoS One. 2025 Jan 13;20(1):e0299781. doi: 10.1371/journal.pone.0299781 (PMC11730379; doi:10.1371/journal.pone.0299781)
Supplement: Attachment — Submitted filename: Response to Reviewers.docx [file pone.0299781.s006.docx]

August 16, 2024

Dr. Jinyi Wu

Academic Editor

PLOS ONE

Dear Dr. Wu,

Thank you for the chance to revise *Sociodemographic and Behavioral Factors Associated with Diet Quality among Low-income Community Health Center Patients with Hypertension*. We are happy that the original reviewer was satisfied with our responses to their original comments. Below we have responded to the additional reviewer’s comments point-by-point. We believe we have satisfied all requirements and thank the reviewers.

Sincerely,

Jessica Cheng, PHD

Department of Epidemiology, Harvard T H Chan School of Public Health

Division of General Internal Medicine, Massachusetts General Hospital

**Response to Reviewers**

Reviewer #2: In this study, the authors used data from a low-income, urban cohort to identify correlates of diet quality among individuals with hypertension. Interventions to improve the health and well-being of underserved populations is important, however, I have some serious concerns about the paper in its present form. This seems like some of the exploratory analysis that would be necessary in informing intervention design, however, I do not think there is enough here for a standalone paper. I am not sure that it adds much to the literature beyond what is already known. I am also concerned about the discussion of certain factors as causal or as a point for intervention (e.g., smoking behavior). While smoking and diet are correlated, there is no causal relationship, and while the authors acknowledge this, they suggest using smoking behavior to inform their intervention anyways. This also has the potential to reinforce stigmas against those the intervention seeks to serve, and so I recommend that the authors consider reframing the discussion around their findings.

*Response: Thank you for this thoughtful suggestion. We have removed any sentences which may have indicated causal relationships between social factors (e.g., smoking) and dietary intake or reinforced stigma (e.g., we have cut the sentence: “Awareness of substance use patterns could help contextualize dietary guidance provided by a CHW and provide an opportunity for discussing the relationship of the substance, such as tobacco or alcohol use, with hypertension.”.*

Additional comments:
The authors mention that NIH/AHA recommend CHW-led interventions (refs. 11,12). Are there any examples of successful CHW-led interventions to improve dietary quality among low-income populations? If so it may be helpful to reference these; if not it may be helpful to explicitly mention that this is a gap that the study team aims to fill.

*Response: This is a helpful suggestion. We note the similarities between our current and planned work as well as how our work expands on that which has already been done: “CHW-delivered hypertension programs are promising ways of reaching diverse participants in consultation with, and outside of, primary care (44, 45). Other CHW-led interventions with dietary components show potential effectiveness in reducing blood pressure and improving health behaviors although diet quality measures, such as the HEI, are not evaluated (46-48). As with other CHW-led hypertension interventions, our planned intervention development and evaluation will include qualitative work to complement our quantitative analyses (46, 49). Thus, we have planned semi-structured interviews with both patients and CHWs, in order to contextualize our current findings and to explore if and how to address these factors in our intervention design and delivery (e.g., involving female partners in the intervention). Thus, our work utilizes similar practices as other promising interventions and expands on existing literature as we will explicitly focus on robust measurement of diet quality outcomes.*

While I appreciate that this is a secondary data analysis, it seems like the best way to determine what sorts of interventions would work well for this population would be to ask the participants directly. I recommend some qualitative work to support the design of this intervention. It would also help contextualize some of the results reported here, such as meal skipping. The reason for meal skipping (income, time constraints, lack of access to appropriate food preparation facilities) is likely important for the participant’s overall diet quality and for the success of the intervention. If qualitative work is planned, I recommend mentioning this explicitly as something that will support the overall intervention design.

*Response: We completely agree with the reviewer’s comment, and we are in the process of conducting qualitative work to build on and complement this analysis. As recommended by the reviewer we add a sentence explicitly acknowledging the value of this qualitative work:* “*As with other CHW-led hypertension interventions, our quantitative analysis should be complemented by qualitative work. Thus, we have planned semi-structured interviews with both patients and CHWs, in order to contextualize our current findings and to explore if and how to address these factors in our intervention design and delivery (e.g., involving female partners in the intervention).”*

Line 60 - A bit confusing why CHWs would seek to prioritize certain factors over the others – particularly for sociodemographic behaviors, but also for behavioral factors as these are correlates but not causal. I recommend cutting this paragraph.

*Response: We have cut this paragraph as recommended.*

Line 79. Much of this data collection took place during the first months of the COVID pandemic. This was a time of unprecedented disruption to people’s lives, the food system, and the healthcare system. There were massive surges in food insecurity in the US during these months. I suggest the authors add a sentence in the limitations acknowledging the potential role of COVID on the data collection.

*Response: Thank you. We have added a sentence to this effect: “Finally, this was a relatively small sample of patients from one urban health system in the northeast US and data collection occurred during the COVID-19 pandemic. Thus, results may not be generalizable to patients from rural areas or from other regions within the US or outside the US, and the pandemic might have temporarily increased the reporting of social needs and/or lead to the worsening of diet quality.”*

Lines 95-96 Please clarify what is meant by “Gender, race, education, marital status, and employment categories were collapsed because of small cell sizes.” It would be helpful if there was a supplemental table that detailed what all categorical options were and how categories were collapsed.

*Response: We have provided analytical code (and formats) on open science framework (DOI 10.17605/OSF.IO/WY4XF) which includes information on the categories before and after collapsing.*

Lines 110-120 are their full lists of what is included in these scales? And are there citations for these measures? Many of the other variables included come from validated questionnaires with citations (e.g., General Anxiety Disorders Questionnaire). It would be helpful if the full response options were provided in a supplement, and if the validations of these measures were cited (where available).

*Response: For the 3-item housing related questions we have added two citations about the development and validation. The other items in this paragraph were created to be relevant to our population and resources. These items on living site problems, receiving housing assistance, receiving transportation assistance, and help obtaining these types of assistance have been added to a supplement. We direct readers in this paragraph to view the supplement. We also note that our analytical code which is available on open science framework (DOI 10.17605/OSF.IO/WY4XF) does provide descriptions of each of these items (i.e., a variable label) as well as for all items included in the analysis. We hope that this is sufficient detail for the understanding of readers.*

Lin 154. Why was HEI the metric of diet quality used and not (for example) DASH, which is mentioned in both the introduction and the discussion as a dietary pattern to address hypertension, or A-HEI, which has stronger associations with BP? I suggest the authors add a sentence justifying the use of HEI as the indicator of diet quality rather than one of these other metrics.

*Response: Thank you. We have added a sentence indicating that the HEI was used as it aligns with the US Dietary Guidelines for Americans. While preferences may differ on which index to use, we find it useful in our work to align with the guidelines as they are familiar to a broad segment of both the research community and the general population: “We chose to use the Healthy Eating Index-2020 (HEI-2020) (28, 29) as the measure of diet quality in this study as it is a valid and reliable measure of overall diet quality aligned with the Dietary Guidelines for Americans.”*

*We have also added a sentence showing that the HEI does relate to blood pressure and is correlated with other indices, specifically DASH and AHEI: “The HEI has been shown to relate to blood pressure, (30, 31) correlate with other diet quality indices, and shows a similar relationship to CVD mortality as other indices (32).”*

The discussion could benefit from discussion of different interventions and policies beyond CHWs to improve diet and hypertension among this population. For example, we know that cash transfers are more impactful in improving diet and health, especially among low-income populations such as that included in this study.

*Response: As the reviewer indicates, the purpose of this analysis was to inform a CHW healthy eating intervention as it is being developed through both quantitative and qualitative means. We believe that additional discussion of the effectiveness of non-CHW nutrition education forms of intervention such as cash transfer programs is beyond the scope of this paper. However, we do agree with the reviewer that cash transfers are a very promising form of intervention for low socioeconomic groups that should be further researched and promoted.*

*To address the role of financial support or incentives as a potential strategy to improve diet, we have added to our discussion: “…connecting patients with community-based nutrition services may help improve the diet of individuals with hypertension as our model suggested that receiving support for food acquisition (i.e., meals at a community organization and receiving a supermarket gift card) was positively associated with diet quality in this mostly low-income population.”*

The authors mention time constraints as a barrier to healthy diet among low-income interventions. How would a CHW-led intervention help to address time constraints experienced by these individuals? Wouldn’t participation in such an intervention just be an additional time pressure?

*Response: Thank you for this comment. Although participating in the CHW program requires time, the goal is to make the intervention as convenient and efficient as possible for the participant. For example, the CHW will provide guidance by phone or video call at a time and place that works for the participant, and therefore this does not require time spent traveling to the clinic for a visit with a dietitian or other clinician.*

*In addition, our goal is that through participating in the CHW program, participants can learn how to address other time constraints specific to shopping and food preparation and that they can use these skills during and after the program has ended. We have added the sentence: “For example…meal planning, and quick healthy go-to meals. We note that while participation in a phone- and web-based CHW -led program is a temporary time constraint while enrolled, we hope that CHWs can help patients develop and/or strengthen skills that reduce the time burden associated with shopping and cooking.”*

Figure 2 – this is a bit hard to read as constructed, I recommend adding additional spacing between the different factors to make it easier to read

*Response: Thank you for this suggestion. We have increased spacing.*
